# Beef embryos in dairy cows: calfhood growth of Angus-sired calves from Holstein, Jersey, and crossbred beef dams

**DOI:** 10.1093/tas/txad096

**Published:** 2023-08-14

**Authors:** Luke K Fuerniss, J Daniel Young, Jerica R Hall, Kaitlyn R Wesley, Oscar J Benitez, Larry R Corah, Ryan J Rathmann, Bradley J Johnson

**Affiliations:** Department of Animal and Food Sciences, Texas Tech University, Lubbock, TX 79409, USA; Department of Animal and Food Sciences, Texas Tech University, Lubbock, TX 79409, USA; Department of Animal and Food Sciences, Texas Tech University, Lubbock, TX 79409, USA; Department of Animal and Food Sciences, Texas Tech University, Lubbock, TX 79409, USA; Department of Animal and Food Sciences, Texas Tech University, Lubbock, TX 79409, USA; Department of Animal Sciences and Industry, Kansas State University, Manhattan, KS 66502, USA; Department of Animal and Food Sciences, Texas Tech University, Lubbock, TX 79409, USA; Department of Animal and Food Sciences, Texas Tech University, Lubbock, TX 79409, USA

**Keywords:** beef, calf, dairy, embryo

## Abstract

Improved reproductive management has allowed dairy cow pregnancies to be optimized for beef production. The objective of this sire-controlled study was to characterize the effects of beef or dairy maternal genetics and the dairy management system on calf growth. Pregnancies were created with a 2 × 2 factorial arrangement of dam breed (Holstein or Jersey) and mating type (artificial insemination or implantation of an in vitro produced embryo from a commercial beef cow oocyte). Resulting calves were reared in a calf ranch. Additionally, commercial beef cows were inseminated and reared resulting calves on range. Therefore, the five treatments were Angus × Holstein (A × H; *n* = 19), Angus × Jersey (A × J; *n* = 22), Angus × beef gestated by Holstein (H ET; *n* = 18), Angus × beef gestated by Jersey (J ET; *n* = 8), and Angus × beef raised by beef (A × B; *n* = 20). Beginning at birth, calf body weight, cannon circumference, forearm circumference, top width, hip width, and hip height were measured approximately every 28 d until ~196 d of age. At birth, A × J calves weighed the least (*P *< 0.01). At 150 d of age, body weight was greatest (*P *< 0.05) among A × B calves, intermediate among H ET and A × H calves, and least among J ET and A × J calves (*P *< 0.05). Morphometric differences were detected between treatments (multivariate analysis of variance, *P *< 0.01). Primary discriminant function scores identified A × B calves having lesser values than A × J or A × H calves (analysis of variance [**ANOVA**], *P* < 0.01); A × B calves had greater cannon circumference, greater top width, and less hip height (standardized loadings of −0.47, −0.48, and 0.63, respectively). Secondary discriminant function scores identified J ET and H ET to have greater forearm circumference—a key indicator of muscling—than A × J or A × H (ANOVA, *P *< 0.01; standardized loading of 0.99). The dairy management system limited growth rate of beef genetics compared to the beef management system. In addition, Holstein dams transmitted greater growth potential than Jersey dams. Replacing maternal dairy genetics with beef genetics moderated frame size and created a more muscular phenotype.

## Introduction

The concept of crossing beef and dairy breeds has been evident for more than 100 yr (Haecker, 1920; Berg and Butterfield, 1968; Ziegler et al., 1971; Cartwright, 1983; Perry et al., 1991). However, recent advances in genomic technologies (Crowe et al., 2021), reproductive efficiency (Cardoso Consentini et al., 2021), and sex-selected semen (Hutchison et al., 2016) have enabled more precise dairy replacement heifer production and widespread use of beef semen on commercial dairies (McWhorter et al., 2020). Recent reviews by Berry (2021), Crowe et al. (2021), Basiel and Felix (2022), Foraker et al. (2022), Poock and Beckett (2022), and Jaborek et al. (2023) emphasize academic interest in beef × dairy crossbred calves. Similarly, selection indexes aimed at optimizing beef semen used in dairy herds by American Simmental Association and American Angus Association summarize interest of the beef industry in the concept of beef × dairy crossbreeding. Since calf value is greater for beef × dairy crossbred calves compared to straightbred dairy calves, beef genetics will likely continue to be used in dairy breeding systems (Cabrera, 2022; McCabe et al., 2022).

The predominantly Holstein dairy herd has increasing Jersey influence because of improved health, reproductive performance, and milk components (Anderson et al., 2007; Heins et al., 2008a, 2008b; Guinan et al., 2019). However, Jersey influence is generally negative for beef production because of poor rate of gain and red meat yield (Lehmkuhler and Ramos, 2008). While differences in calf, feedlot, and carcass performance, are evident between beef and dairy breeds (Ziegler et al., 1971; Perry et al., 1991; Thonney et al., 1991; Abney, 2004), it is difficult to control comparisons of beef and dairy calves since management system confounds breed type in most production scenarios. When beef and beef × dairy calves are managed under the same conditions on a calf ranch, differences in growth and muscularity could be less than observed under typical management. Therefore, the objectives of this experiment were 1) to use an embryo transfer model to investigate the effect of the dairy management system on beef genetics and 2) to directly compare the maternal contributions of Holstein, Jersey, and Angus-based beef cows to terminally relevant traits among Angus-sired calves.

## Materials and Methods

### Animal Care and Use

All calves born and reared at Texas Tech University were managed according to protocol 19048-05 and SOP022 approved by Texas Tech University Animal Care and Use Committee. All calves born and reared on commercial dairies were managed in accordance with *The Guide for Care and Use of Agricultural Animals in Research and Teaching.*

### Mating System Design

Calves enrolled in this study were sired by a single Angus bull (GAR Momentum, American Angus Association registration number 17354145). At the time of trial initiation, GAR Momentum’s expected progeny differences ranked in the best 5% of the breed for calving ease direct, marbling (**Marb**), ribeye area (**RE**), beef value index ($B), Angus-on-Holstein index ($A × H), and Angus-on-Jersey ($A × J) index. Three cow herds were used to produce calves for this study: 1) crossbred beef cows managed on range in New Deal, TX, 2) Holstein cows managed on a commercial dairy located 195 km southwest of New Deal, TX, 3) Jersey cows managed on a commercial dairy in 190 km southwest of New Deal, TX. Beef cows were managed representatively of commercial cow/calf production in the United States; Holstein and Jersey cows were managed representatively of commercial dairy production in the United States.

Both artificial insemination and embryo transfer were used to create pregnancies. Embryos were produced by in vitro fertilization (SimVitro HerdFlex; J. R. Simplot Company; Boise, ID). Oocytes were aspirated from ovaries of commercial black, polled, Angus-based beef cows immediately after slaughter. Semen from GAR Momentum was used to fertilize oocytes. After incubation and maturation, grade 1 embryos were frozen in 0.25 mL straws in media suitable for direct transfer of thawed embryos for later use.

Matings occurred over an 85-d period beginning in mid-May of 2020. Crossbred beef, Holstein, and Jersey cows were inseminated with GAR Momentum semen. Additionally, embryos (composed of straightbred beef genetics; GAR Momentum × commercial beef cow) were transferred to Holstein and Jersey recipient females. The combinations of dam breed and mating type were the five treatments considered in this study. The five treatments were as follows: 1) Angus-sired beef calves managed through the beef production system (A × B, *n = *20), 2) Angus-sired beef calves born to Holstein dams and managed through the dairy production system (**H ET**, *n = *18), 3) Angus × Holstein crossbred calves managed through the dairy production system (A × H, *n = *19), 4) Angus-sired beef calves born to Jersey dams and managed through the dairy production system (**J ET**, *n = *8), 5) Angus × Jersey crossbred calves managed through the dairy production system (A × J, *n = *22).

### Enrollment of Calves

Calves were identified for enrollment shortly after birth. Only calves with confirmed mating records were considered for the study. More pregnancies were created by artificial insemination than needed for a balanced design. Therefore, A × H and A × J calves were selected for enrollment based on birth of H ET and J ET calves, respectively. When a calf from an embryo transfer mating was born (H ET and J ET), the calf was enrolled in the study and a calf born from an artificial insemination mating of the same dam breed (A × H and A × J) was also enrolled. Criteria for enrolling a calf from an artificial insemination mating included birthdate within 1 d and matching sex to the embryo transfer calf. Additional A × J calves were enrolled in the study beyond the number of J ET calves enrolled; A × J calves were selected to match the average birthdate of the J ET calves.

### Beef Production System

Dams of A × B calves were managed on rangelands with no less than 1.29 hectares per cow. Free-choice mineral was offered. Supplemental hay (*Bothriochloa bladhii*) was provided from calving through ~30 d postpartum. Within 24 h of birth, calves were given individual ID tags and umbilical cords were sprayed with 7% iodine solution. Calves were vaccinated intranasally against bovine respiratory syncytial virus, infectious bovine rhinotracheitis virus, and parainfluenza 3 virus (Inforce3; Zoetis, Parsippany, NJ) and vaccinated orally against bovine rotavirus and coronavirus (Calf-Guard, Zoetis). Calves were also injected with vitamins A, D, and E (Vitamin E-AD Injection; VetOne, Boise, Idaho) and trace minerals (MultiMin 90; MultiMin North America, Fort Collins, CO). Supplemental perinatal warmth was provided as needed. At ~60 d of age, bull calves were nonsurgically castrated, and all calves were vaccinated against *Clostridium* (Bar-Vac CD/T; Boehringer Ingelheim, Ridgefield, CT). At ~90 d of age, all calves were vaccinated against respiratory pathogens (Bovi-Shield Gold 5; Zoetis). At ~6 mo of age, all calves were vaccinated and then revaccinated at ~7 mo of age against respiratory (Bovi-Shield Gold 5; Zoetis; and Bovilis Once PMH IN; Merck, Rahway, NJ) and clostridial diseases (Bovilis Vision 7 Somnus with Spur; Merck) in preparation for comingling and feedlot entry. Calves remained with their dams for the duration of this study.

### Dairy Production System

Calves born in the dairy production system (A × H, A × J, H ET, and J ET) were separated from their dams at birth. Each calf was given an individual ID and its umbilical cord was sprayed with 7% iodine solution. Calves were vaccinated intranasally against bovine respiratory syncytial virus, infectious bovine rhinotracheitis virus, and parainfluenza 3 virus (Inforce3; Zoetis). In addition to 3.78 L of pasteurized colostrum, calves were given oral antibodies to protect against *Escherichia coli* and coronavirus infection (FirstDefense; ImmuCell Corporation, Portland, ME). Calves were housed individually in plastic hutches with outdoor pens. Calves were offered 2.84 L of milk twice per day. Milk consisted of pasteurized whole milk adjusted to 13% dry matter with supplemental 24% protein and 12% fat milk replacer (Calva Products; Acampo, California). All calves were offered free choice access to water and textured starter feed (composition in Table 1). At ~55 d of age, calves were vaccinated against *Moraxella bovoculi* (*Moraxella bovoculi* Bacterin; Merck).

**Table 1.** Chemical composition of diets^1^^,^^2^

	Diet		
	Starter	Phase I grower	Phase II grower
Dry matter, %	87.0	73.7	61.1
Crude protein, %	22.1	15.7	12.5
Ether extract, %	3.1	3.4	2.7
ADF, %	8.6	16.4	26.9
NDF, %	19.7	26.6	41.3
NE_M_, Mcal/kg	2.31	1.93	1.50
NE_G_, Mcal/kg	1.61	1.28	0.90

^1^All values except DM expressed on dry-matter basis.

^2^Samples that were composited and analyzed at a commercial laboratory (ServiTech Laboratories, Amarillo, TX).

At ~65 d of age, calves were weaned and offered the same starter feed with group housing (approximately *n* = 8 calves per pen). Bull calves were nonsurgically castrated at ~75 d of age; all calves were vaccinated against respiratory viruses, *Leptospira*, and *Camplylobacter* (Bovilis Vista 5 L5 SQ CFP; Merck) and *Clostridium* (Alpha-7/MB-1; Boehringer Ingelheim). At ~85 d of age, calves were moved to large group pens and revaccinated against *Moraxella bovoculi* (*Moraxella bovoculi* Bacterin; Merck). For the first ~28 d, calves were offered phase I grower diet ad libitum; for the rest of the study, calves were offered phase II grower diet ad libitum (composition in Table 1). Both grower diets were fed as a total mixed ration and included barley silage, corn gluten feed, ground corn, cotton burrs, and supplement providing vitamins and minerals. At ~6 mo of age, all calves were vaccinated and revaccinated at ~7 mo of age against respiratory (Bovi-Shield Gold 5; Zoetis; and Bovilis Once PMH IN; Merck) and clostridial diseases (Bovilis Vision 7 Somnus with Spur; Merck) in preparation for comingling and feedlot entry.

### Body Measurements

All calves were measured from birth to ~196 d of age in 28 d intervals. In total, calves were sampled eight times throughout the study. Calves in the beef production system were first sampled within 24 h of birth. The second sampling occurred when the average age of the A × B calves was 28 d. Third through eighth samplings occurred in 28-d intervals following the previous sampling. Calves in the dairy production system were first sampled within 72 h of birth. The second through fourth samplings occurred in 28-d intervals following the previous sampling. The time of the fifth sampling was determined based on group average age of 112 d with calves born to Holstein dams (A × H and H ET) sampled separately from calves born to Jersey dams (A × J and J ET). The sixth, seventh, and eighth samplings occurred in 28-d intervals following the previous sampling.

At each sampling, BW and body measurements were recorded. Body weight was measured by a hanging scale with a sling and recorded to the nearest 0.45 kg at birth; at subsequent samplings, BW was measured using a platform scale. Additional body measurements were obtained at each sampling using similar methods to those described by Tatum et al. (1986b). Measurements were recorded to the nearest tenth of a centimeter and included the following:

1. Cannon circumference: circumference of the left forelimb measured using flexible tape at the point of least circumference distal to the carpal joint and proximal to the metacarpophalangeal joint.
2. Forearm circumference: circumference of the left forelimb measured using flexible tape immediately distal to the lateral epicondyle of the humerus.
3. Top width: distance measured using caliper between the most lateral tissues at the first lumbar vertebra.
4. Hip width: distance measured using caliper between the lateral surfaces of the tuber coxae.
5. Hip height: distance measured using caliper-type device from a point on the dorsal midline between the tuber coxae to the chute floor while the animal was standing with natural posture.
6. Body length: distance measured using caliper-type device parallel to animal’s back between lateral tuberosity of the humorous and the ischiatic tuberosity.

All described measurements were recorded at each sampling time point except hip height which was not measured at birth.

### Statistical Analysis

Univariate analyses were conducted to test differences between treatment groups at birth, 60 d, and 150 d of age. Body weights were analyzed unshrunk, and all response variables were log-transformed to approximately satisfy the assumption of normality. Linear models were fit using the lme4 package of R (Bates et al., 2015) and included slope and intercept terms for each treatment, sex as a fixed effect, and animal as a random effect to account for repeated measurement (and lack of independence). The model for birth included measurements from first and second samplings for each calf; the model for 60 d of age included measurements from second through fourth samplings for each calf (~28, 56, and 84 d of age); the model for 150 d of age included measurements from fourth through eighth samplings for each calf (~84, 112, 140, 168, and 196 d of age). If a calf had missing data from a sampling timepoint (e.g., died) that calf was removed from the model. Standard error of the mean was calculated separately for each treatment and estimated marginal means were calculated using the emmeans package of R (Lenth, 2022). Significance was assessed by ANOVA testing of the main effect of treatment group; pairwise comparisons were protected by ANOVA significance, and Bonferroni adjustment was used for multiple comparisons.

Multivariate analyses were conducted to test morphometric differences between treatment groups at a common unshrunk BW of 136 kg. Body measurements for each calf from its sampling closest to 136 kg were adjusted to 136 kg by linear models. All observations from which a calf weighed between 90.7 and 181.4 kg were included to build models to predict body measurements from BW. Linear models were fit using the lme4 package of R (Bates et al., 2015) and included slope and intercept terms for each treatment and animal as a random effect to account for repeated measurement. Slope coefficients were used to adjust each calf’s body measurements to BW of 136 kg. Adjusted data were tested for univariate outliers (by a range of 1.5 multiplied by the interquartile range added to the third quartile and subtracted from the first quartile) and multivariate outliers (Mahalanobis, 1936). Normality was assessed by Shapiro–Wilk (Shapiro and Wilk, 1965) testing, and homogeneity of variance was assessed by Levene’s test (Levene, 1960) and Box M testing (Box, 1949). Multicollinearity was assessed, and body length observations were removed because of correlation with hip height (*r* = 0.48). Multivariate analysis of variance (**MANOVA**) with Wilk’s test statistic was used to test for treatment differences with Bonferroni-adjusted multiple comparisons completed using the Biotools package of R (da Silva et al., 2017). Discriminant function analysis was completed using the MASS package of R (Venables and Ripley, 2002). Loadings were extracted and standardized by the methods of Coghlan (2017). Axis scores of discriminant functions were tested by one-way ANOVA with Bonferroni-adjusted pairwise comparisons.

Based on discriminant function analysis, a model was constructed to predict unshrunk BW from measured hip width for calves born on dairies (A × H, A × J, H ET, and J ET). The relationship between hip width and weight was modeled on a log–log scale and back transformed to represent the relationship as a power equation. Mixed models were fit using the lme4 package of R (Bates et al., 2015) and included individual animal in the model as a random effect. Marginal and conditional coefficients of determination were calculated using the MuMIn package of R (Barton, 2022).

All statistical analysis was performed in R version 4.2.1 (R Core Team, 2022). Statistical significance was evaluated compared with α of 0.05. When 0.05 < *P* ≤ 0.10, tendencies were considered. Visualizations were built in R using the ggplot2 package (Wickham, 2016) and ggpubr package (Kassambara, 2020).

## Results

### Body Weight

At birth, all calves were similar in BW except for A × J calves which had BW 15% less than the average of all other groups (*P* < 0.05; Table 2). Regardless of mating type (artificial insemination or embryo transfer) or gestational dam breed (beef, Holstein, or Jersey), straightbred beef calves (A × B, H ET, and J ET) had similar BW at birth (*P* > 0.05). By 60 d of age, BW of calves in the beef production system (A × B) was 56% greater (38.6 kg) than the average BW of calves born in the dairy production system (*P* < 0.05; Table 3). The BW of calves gestated by a Holstein cow (A × H or H ET) was 24% greater than the BW of calves gestated by a Jersey cow (A × J or J ET) at 60 d of age (*P* < 0.05). Similarly, BW of calves gestated by a Holstein cow (A × H or H ET) was 27% greater than calves gestated by a Jersey cow (A × J or J ET) at 150 d of age (*P* < 0.05; Table 4). By 150 d of age, the BW of calves in the beef production system (A × B) was 45% greater (63.0 kg) than the average BW of calves born in the dairy production system (*P* < 0.05). At 196 d of age, A × B calves weighed 262 ± 7.4 kg.

**Table 2.** Estimated marginal means^1^ and standard errors^2^ of body measurements at birth

	Treatment^3^					*P* value^4^
	A × B	H ET	A × H	J ET	A × J	
*n*	20	18	19	8	22	
*n*, male	12	6	6	7	13	
*n*, female	8	12	13	1	9	
BW, kg	37.4^a^ ± 1.01	37.4^a^ ± 1.10	37.6^a^ ± 1.13	39.3^a^ ± 1.99	32.3^b^ ± 1.04	<0.01
Cannon circumference, cm	11.9^a^ ± 0.16	11.4^ab^ ± 0.17	10.8^b^ ± 0.16	10.7^bc^ ± 0.28	9.9^c^ ± 0.16	<0.01
Forearm circumference, cm	24.7^a^ ± 0.42	24.0^a^ ± 0.48	22.0^b^ ± 0.45	22.6^ab^ ± 0.84	19.3^c^ ± 0.47	<0.01
Top width, cm	11.0^a^ ± 0.17	10.6^ab^ ± 0.19	10.2^b^ ± 0.19	10.2^ab^ ± 0.34	10.0^b^ ± 0.22	<0.01
Hip width, cm	15.6^a^ ± 0.20	15.7^a^ ± 0.24	15.2^ab^ ± 0.24	15.9^a^ ± 0.45	14.5^b^ ± 0.27	<0.01
Body length, cm	61.9 ± 0.83	61.8 ± 0.99	61.1 ± 1.03	61.9 ± 1.91	61.1 ± 1.24	0.96

^1^Adjusted to 0 d of age by back transforming a logarithmic regression model of first and second sampling for each calf (~0 and 28 d of age) including slope and intercept terms for each treatment with sex as a fixed effect and the individual as a random effect.

^2^Standard error of the mean calculated separately for each treatment.

^3^Treatments included Angus-sired beef calves (A × B), Angus-sired beef calves born to Holstein dams (H ET), Angus × Holstein crossbred calves (A × H), Angus-sired beef calves born to Jersey dams (J ET), Angus × Jersey crossbred calves (A × J). A × B calves were managed in a beef production system; all other calves were managed in a dairy production system.

^4^Presented as the ANOVA significance for the main effect of treatment.

^a,b,c^Means without common superscript differ (*P *< 0.05) by pairwise comparisons.

**Table 3.** Estimated marginal means^1^ and standard errors^2^ of body measurements at 60 d of age

	Treatment^3^					*P* value^4^
	A × B	H ET	A × H	J ET	A × J	
*n*	20	17	19	8	22	
*n*, male	12	6	6	7	13	
*n*, female	8	11	13	1	9	
BW, kg	107.7^a^ ± 2.67	74.4^b^ ± 1.97	78.4^b^ ± 2.02	64.3^c^ ± 2.58	59.2^c^ ± 1.4	<0.01
Cannon circumference, cm	14.0^a^ ± 0.14	12.6^b^ ± 0.14	12.5^b^ ± 0.13	12.5^b^ ± 0.21	11.5^c^ ± 0.11	<0.01
Forearm circumference, cm	31.4^a^ ± 0.38	28.7^b^ ± 0.37	27.8^b^ ± 0.35	29.1^b^ ± 0.57	26.2^c^ ± 0.30	<0.01
Top width, cm	17.1^a^ ± 0.18	14.2^b^ ± 0.16	14.0^b^ ± 0.15	13.7^b^ ± 0.23	12.8^c^ ± 0.13	<0.01
Hip width, cm	23.2^a^ ± 0.23	20.8^bc^ ± 0.22	21.2^b^ ± 0.21	20.0^c^ ± 0.31	19.0^d^ ± 0.18	<0.01
Hip height, cm	79.1 ± 0.55	75.3 ± 0.56	80.0 ± 0.58	74.0 ± 0.83	75.1 ± 0.50	0.10
Body length, cm	84.7 ± 0.68	80.2 ± 0.69	82.2 ± 0.68	77.6 ± 1.00	76.3 ± 0.58	0.14

^1^Adjusted to 60 d of age by back transforming a logarithmic regression model of second through fourth sampling for each calf (~28, 56, and 84 d of age) including slope and intercept terms for each treatment with sex as a fixed effect and the individual as a random effect.

^2^Standard error of the mean calculated separately for each treatment.

^3^Treatments included Angus-sired beef calves (A × B), Angus-sired beef calves born to Holstein dams (H ET), Angus × Holstein crossbred calves (A × H), Angus-sired beef calves born to Jersey dams (J ET), Angus × Jersey crossbred calves (A × J). A × B calves were managed in a beef production system; all other calves were managed in a dairy production system.

^4^Presented as the ANOVA significance for the main effect of treatment.

^a,b,c,d^Means without common superscript differ (*P *< 0.05) by pairwise comparisons.

**Table 4.** Estimated marginal means^1^ and standard errors^2^ of body measurements at 150 d of age

	Treatment^3^					*P* value^4^
	A × B	H ET	A × H	J ET	A × J	
*n*	19	16	19	5	17	
*n*, male	11	6	6	4	10	
*n*, female	8	10	13	1	7	
BW, kg	202.8^a^ ± 5.54	152.7^b^ ± 4.56	160.6^b^ ± 4.56	124.9^c^ ± 5.71	121.1^c^ ± 3.19	<0.01
Cannon circumference, cm	15.8^a^ ± 0.15	14.7^b^ ± 0.15	14.7^b^ ± 0.15	14.1^b^ ± 0.24	13.1^c^ ± 0.12	<0.01
Forearm circumference, cm	39.7^a^ ± 0.44	38.6^ab^ ± 0.46	38.3^ab^ ± 0.44	36.5^b^ ± 0.71	33.2^c^ ± 0.36	<0.01
Top width, cm	21.9^a^ ± 0.29	19.2^b^ ± 0.28	18.9^b^ ± 0.26	17.4^c^ ± 0.40	16.8^c^ ± 0.22	<0.01
Hip width, cm	30.3^a^ ± 0.32	27.8^b^ ± 0.32	28.3^b^ ± 0.31	26.0^c^ ± 0.48	25.2^c^ ± 0.26	<0.01
Hip height, cm	100.2^a^ ± 0.80	91.8^b^ ± 0.79	99.8^a^ ± 0.83	88.8^b^ ± 1.23	90.5^b^ ± 0.71	<0.01
Body length, cm	107.5^a^ ± 0.84	100.0^b^ ± 0.85	102.5^b^ ± 0.83	94.2^c^ ± 1.27	95.5^c^ ± 0.73	<0.01

^1^Adjusted to 150 d of age by back transforming a logarithmic regression model of fourth through eighth sampling for each calf (~84, 112, 140, 168, and 196 d of age) including slope and intercept terms for each treatment with sex as a fixed effect and the individual as a random effect.

^2^Standard error of the mean calculated separately for each treatment.

^3^Treatments included Angus-sired beef calves (A × B), Angus-sired beef calves born to Holstein dams (H ET), Angus × Holstein crossbred calves (A × H), Angus-sired beef calves born to Jersey dams (J ET), Angus × Jersey crossbred calves (A × J). A × B calves were managed in a beef production system; all other calves were managed in a dairy production system.

^4^Presented as the ANOVA significance for the main effect of treatment.

^a,b,c^Means without common superscript differ (*P *< 0.05) by pairwise comparisons.

### Body Measurements Relative to Age

From birth through 150 d of age, A × B calves were consistently the largest measuring while A × J calves were consistently the smallest measuring (Tables 2, 3, and 4). Cannon circumference of A × J calves was the least at all timepoints (*P* < 0.05) and was consistently less than the cannon circumference of A × B calves by 20% to 21%. While cannon circumference of A × H, H ET, and J ET calves were similar to the cannon circumference of A × B calves at birth (*P* > 0.05), A × H, H ET, and J ET calves had lesser cannon circumference by 10% and 8% at 60 and 150 d of age (*P* < 0.05). At birth, straightbred beef calves had the greatest forearm circumference (A × B, H ET, and J ET). Within Holstein dams, H ET calves had greater forearm circumference than A × H by 9% (*P* < 0.05). Within Jersey dams, J ET calves had greater forearm circumference than A × J by 17% (*P* < 0.05). At 60 and 150 d of age, forearm circumference of A × J remained lesser than that of J ET (*P* < 0.05) while forearm circumference of A × H was only numerically lesser than that of H ET (*P* > 0.05).

Top and hip width measurements exhibited less variation between treatments than circumference measurements. Longitudinal changes in hip width measurements were similar to changes in BW. At birth A × J calves measured with least hip width being lesser by 5% compared to A × H calves (*P* < 0.05) and by 8% compared to straightbred beef calves (*P* < 0.05). Progressing to 150 d of age, A × B calves had the greatest hip width by 13% compared to the average of all other calves (*P* < 0.05). Calves born to a Holstein cow (H ET or A × H) had 10% greater hip width than calves born to Jersey cows (J ET or A × J; *P* < 0.05). Similar to hip width, changes in top width matched longitudinal changes in BW. By 150 d, the A × B calves had the greatest top width, calves born to Jersey cows had the least top width, and calves born to Holstein cows had intermediate top width (*P* < 0.05). Within gestational dam breed, straightbred beef calves had numerically greater top width than calves of half dairy genetics (*P* > 0.05). Top width of H ET calves ranged from 1% to 4% greater than that of A × H calves from birth to 150 d of age. Similarly, top width of J ET calves ranged from 2% to 7% greater than that of A × J calves from birth to 150 d of age.

Though seemingly similar measurements, different patterns were observed for longitudinal changes in body length and hip height. At birth, no differences were observed in body length (*P* = 0.96), but treatment differences approached a tendency by day 60 (*P *= 0.14). At day 150, differences in body length were similar to differences in BW where body length of A × B calves was the longest, body length of J ET and A × J was shortest, and body length of H ET and A × H was intermediate (*P* < 0.05). For hip height, group differences approached tendency but were not statistically different at 60 d of age (*P* = 0.10). However, A × B and A × H had 11% greater hip height than all other calves by 150 d of age (*P* < 0.05). Even though A × B and A × H calves had similar hip height, A × H calves were 50.1 kg lighter (*P* < 0.05). Interestingly, A × H and H ET calves were similar in weight, but A × H calves had a greater hip height by 8.0 cm at 150 d of age (*P* < 0.05).

### Body Measurements Adjusted to Common Weight

**Figure 1.**
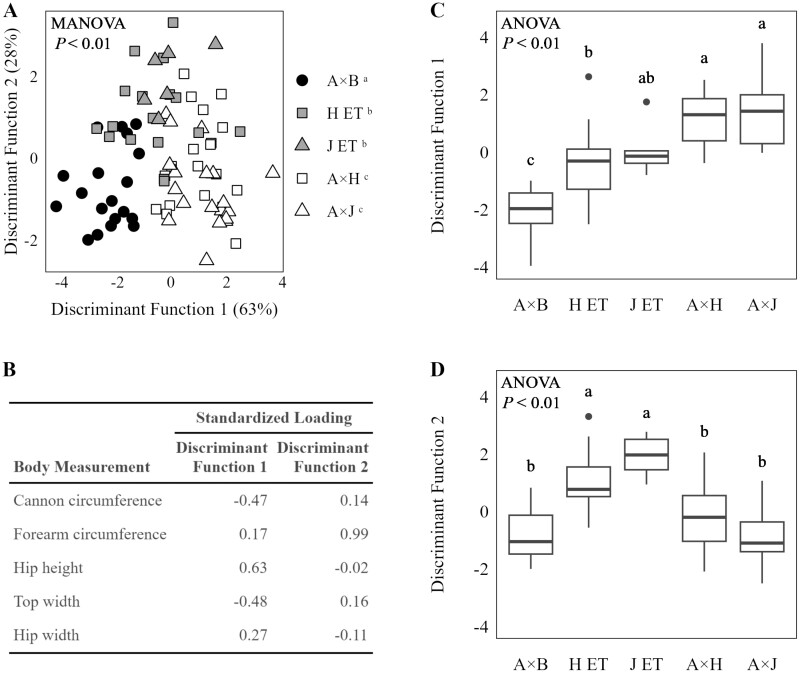
(A) Linear discriminant analysis of body dimension of calves in beef and dairy production systems adjusted to 136 kg of BW. Treatments included Angus-sired beef calves (A × B), Angus-sired beef calves born to Holstein dams (H ET), Angus × Holstein crossbred calves (A × H), Angus-sired beef calves born to Jersey dams (J ET), Angus × Jersey crossbred calves (A × J). A × B calves were managed in a beef production system; all other calves were managed in a dairy production system. (B) Standardized loadings for each body measurement. (C) Testing of scores generated by first discriminant function. (D) Testing of scores generated by second discriminant function. Groups without a common superscript (^a,b,c^) differ by multivariate (A) or univariate (C and D) pairwise comparisons when compared with α = 0.05.

When adjusted to common BW of 136 kg, multivariate differences in body weight were detected by MANOVA testing (*P* < 0.01; Fig. 1). Based on multivariate pairwise comparisons, H ET and J ET were different from A × H and A × J, and A × B was unique from all other treatment groups (*P* < 0.05). Discriminant function analysis was used to identify variables that explained the group differences; axis scores and loadings were visualized in Fig. 1. The first and second discriminant functions accounted for 91% of the variation between treatment groups. Based on the first discriminant function, A × B calves had greater top width and cannon circumference (standardized loadings of −0.48 and −0.47, respectively) while calves of half dairy genetics had greater hip height (standardized loading of 0.63). Straightbred beef calves born to dairy cows (H ET and J ET) were intermediate. The second discriminant function indicated that H ET and J ET had greater forearm circumference (standardized loading of 0.99) than all other treatments. Of all body measurements, hip width explained the least variation between groups when adjusted to a common weight as measured by standardized loadings of 0.27 and −0.11 for the first and second discriminant functions.

### Predicting Body Weight from Hip Width

**Figure 2.**
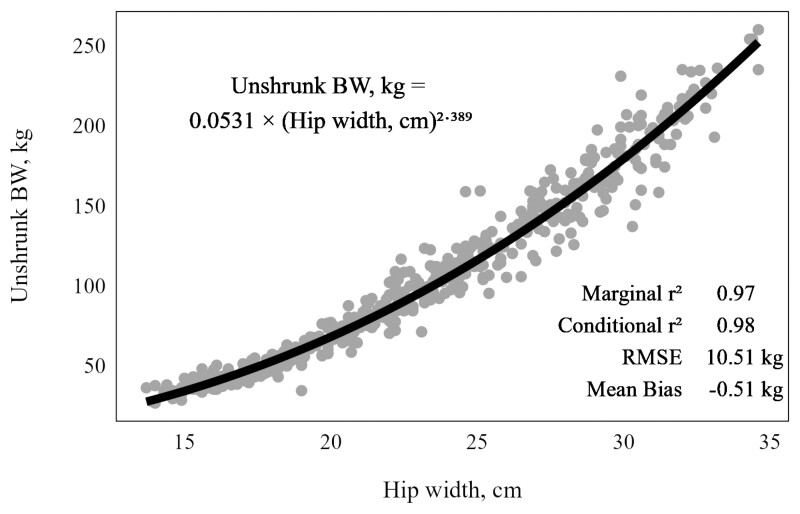
Relationship of hip width and unshrunk BW of Angus-sired calves from Holstein, Jersey, or crossbred beef maternal genetics raised in the dairy production system. Model was fit on a log–log scale with 506 total observations and included fixed effect of hip width and random effect of individual calf (*n *= 68).

The model fit to predict unshrunk body weight from hip width of calves raised in the dairy management system was summarized in Fig. 2. On a log–log scale, the relationship between hip width and BW was described by a linear model with an estimated intercept of −2.936 ± 0.0519 and estimated slope of 2.389 ± 0.0165. Though fit on a log–log scale, the model was presented after being back transformed. The marginal *r*^2^ of 0.97 and the conditional *r*^2^ of 0.98 indicated that the random effect of individual animal (*n* = 68) accounted for ~1% of the variation in BW explained by the model. Most of the variation in BW (97%) was explained by hip width. The mean absolute prediction error was 7.3 kg, and the mean bias was negative 0.51 kg. Root mean square error was 10.51 kg, and the model coefficient of variation was 10.16.

## Discussion

### Calf Performance Relative to Industry Standards

Growth performance of A × B calves was similar to expectations of typical United States cow–calf production systems. Based on the results of a survey completed in 2017, United States Department of Agriculture reported an average weaning weight of 248 kg at an average age of 196 d (USDA, 2020). The beef-type calves in the present study weighed 262 kg at the same age, suggesting that the combination of genetics and management were similar to commercial beef production in the United States. Others have demonstrated that calves of all dairy-type genetics can wean at similar weights when managed alongside their dam on range land. For example, Plum and Harris (1971) found that Holstein calves raised by Holstein dams had an adjusted weaning weight of 240 kg when summered on pasture. Milk production of the dam breed, nutritional quality of pasture, over 50 yr of genetic selection, and many other factors differ between this example and the present study; however, there is potential that calves of dairy genetics could have similar performance to calves of beef genetics when managed under similar conditions.

The dairy production system used in this study was similar to the assumptions used in previous modeling efforts where calves were weaned at 60 d and then transitioned to concentrate feeds (Stackhouse-Lawson et al., 2012). Additionally, the volume of milk offered per feeding was similar to the U.S. commercial calf feeding industry in which 74% of operations feed between 1.9 and 2.8 L of milk or milk replacer per feeding (USDA, 2011; Machado and Ballou, 2022). Preweaning growth of beef and beef cross calves born to Holstein dams (H ET and A × H) was similar to the results of Orellana Rivas et al. (2022) who found ADG of 0.60 to 0.65 kg/d until 63 d among Holstein calves feed 0.65 to 0.76 kg of milk dry matter per day. However, growth performance was lesser than that reported by Zimpel et al. (2021) who found ADG of 0.77 to 0.81 kg/d until d 56 among Holstein calves and Welk et al. (2022) who found ADG of 0.70 to 0.88 kg/d until 70 d. The calves feed by Zimpel et al. consumed ~0.35 kg more milk dry matter per day than was offered to the calves in the present study, and the calves fed by Welk et al. were offered twice the volume of milk offered to the calves in the present study. Growth of the calves in the present study was likely limited by quantity of milk dry matter offered in the first 2 mo of life. Preweaning calf growth could have been accelerated by increasing preweaning plane of nutrition. Since energy exceeding maintenance requirements was limited by the preweaning plane of nutrition, differences in growth potential of H ET and A × H could have been masked.

Consistently from birth to 150 d of age, the A × J calves had the lightest BW which is well supported by differences in mature size of Jersey, Holstein, and Angus-crossbred cows. Mature size of Jersey cows is generally more than 100 kg less than that of Holstein cows or commercial beef cows in the US (Rastani et al., 2001; Anderson et al., 2007; Edwards et al., 2017; Bir et al., 2018). Correspondingly, Ware et al. (2015) and Macleod et al. (1970) observed greater body weight and rate of gain for Holstein compared to Jersey calves. Smaller mature size and associated lesser daily gain likely explain the A × J calves having the lightest BW compared to all other calves.

Beef × dairy calves are expected to weigh 160 kg at 150 d of age (Machado and Ballou, 2022). Of the calves raised on the dairy, the only treatment group to achieve this expectation was the A × H calves. While limited published data has evaluated postweaning plane of nutrition in beef × dairy calves, the diets used in this study likely limited calf growth performance. The phase II grower fed in this study had lesser protein and greater NDF than typical ranges reported by Akins (2016) and lesser energy concentration than reported by Rosadiuk et al. (2021). Performance observed in this study should be considered minimal relative to opportunity for postweaning growth.

While calf genetics and mature size offer reason for the A × J calves to be lighter in BW, growth potential of the H ET and J ET calves should have been comparable to the A × H calves. The H ET calves were similar in BW to the A × H calves, but the J ET calves were lighter by almost 30 kg. The comparatively poorer performance of the J ET calves compared to the H ET calves could be partly explained by poorer health of the J ET calves. From birth to 150 d of age, only 5 of 8 J ET calves survived whereas 16 of 18 H ET calves survived. This study was not designed to test morbidity and mortality traits, and small, unbalanced sample sizes make statistical comparison difficult. However, the number of calves surviving to 150 d of age seems to validate visual observation of poorer digestive and respiratory health among J ET calves. When an environment presents health challenges, the H ET and J ET calves are likely at a disadvantage compared to the A × H and A × J calves: compared to beef × dairy matings, heterosis is minimized by the embryo matings in which Angus semen was used to fertilize oocytes from Angus-based cows. Heterosis is particularly impactful on traits related to survivability and rapid growth early in life (Cundiff, 1970, 1977; Smith et al., 1976). Maximal difference in biological type theoretically maximizes heterosis such as in a cross between beef and dairy breeds (Smith, 1976). Heterosis and its implications on calf health and, consequently, growth performance will remain an important factor in discussion of beef × dairy crossbreeding programs.

While the A × B calves had greater BW relative to the calves raised in a dairy production system, a broad conclusion should not be made that beef genetics have greater growth potential than dairy genetics. Such a comparison would be confounded by the management system. Compared to the dairy management system, the beef management system likely supplied more of each calf’s nutrient requirements from milk. During this study, A × B calves had access to their dam and their milk supply for the duration of the study whereas all calves born on a dairy were offered milk for only 65 d. In addition to duration of time offered milk, A × B calves also likely consumed milk in a greater number of meals per day. Odde et al. (1985) reported five suckling events per day when calves were raised with their dam. However, the dairy management system used in this study only offered milk twice daily. In addition, the quantity of milk offered to calves raised on the dairy in this study was ~6 kg. Calf capacity for milk consumption has been estimated at ~8.5 kg/d for the first 40 d (Jasper and Weary, 2002) and ~12 kg/d for calves at 3 mo of age (Plum and Harris, 1971). Milk production of cows similar in age and breed composition to the dams of the A × B calves was between 6 and 12.7 kg/d (Edwards et al., 2017). Based on greater days offered milk, assumed greater frequency of milk consumption, and assumed greater quantity of milk consumed per day, A × B calves likely consumed greater total nutrients from milk, a factor that could have enabled greater quantity of energy to be partitioned for growth. This could explain the lesser performance of H ET and J ET—straightbred beef calves raised in the dairy management system—compared to A × B calves. However, the effects of plane of nutrition alone cannot be separated from other differences in this experimental design such as in vitro mating, embryo transfer, nutritional status of the gestating dam, housing type, and exposure to different microbiota.

### Measures that Differentiate Beef and Dairy Genetics

Across both the beef and dairy management systems, measurement of top width and hip height were valuable for differentiating the phenotypes of calves of straightbred beef and beef × dairy genetics at a common weight. Generally, straightbred beef calves (A × B, H ET, and J ET) had greater width of top and lesser hip height. Beef genetics compared to dairy genetics were hypothesized to express greater muscularity and more moderate frame size. Tatum et al. identified that feeder cattle identified as larger framed by visual assessment had greater measured hip height (1986b) and lesser separable fat at a constant carcass weight (1986a). Based on the work of Tatum et al., the differences in hip height measured in the current study are likely associated with differences in frame size that could affect feedlot performance and carcass composition. Based on comparison at a common weight, Angus-sired calves from Holstein or Jersey dams appear to be taller in stature and larger framed than Angus-sired calves from crossbred beef dams.

Similarly, measures of body width are associated with muscling. Tatum et al. (1986b) found positive correlations between visually assessed muscularity and both width of stifle and width of hip. When cattle were visually assessed as more muscular, greater muscle to bone ratio was identified when carcasses were adjusted to a common bone mass (Tatum et al., 1986a). While top width was not measured by Tatum et al., top width similarly measures skeletal width and is likely associated with greater muscularity. Applied to the present study, straightbred beef calves were more muscular than dairy-influenced calves at a common BW. Another measurement used by Tatum et al. to identify more muscular cattle was forearm circumference. Forearm circumference was greater among J ET and H ET calves compared to A × J and A × H calves.

However, forearm circumference of A × B calves was lesser compared to J ET and H ET when adjusted to a common weight. This was likely caused by differences inherent in the management systems used in this study that resulted in claves reaching 136 kg at different ages. At a common weight, A × B calves were younger than calves born on the dairy caused by greater rate of gain. Additionally, A × B calves had greater opportunity to consume energy from both milk and relatively low-quality standing forage. Access to forage has increased thickness of the rumen wall and associated smooth muscle (Beiranvand et al., 2014), increased the empty weight and volume of the rumen and reticulum (Stobo et al., 1966; Khan et al., 2011), and increased gut fill as a proportion of BW (Jahn et al., 1970, 1976). Previous findings supported the assertion that the beef management system caused A × B calves to have a greater proportion of weight associated with the gastrointestinal tract. If A × B calves were compared to the calves from the dairy management system in this study on a basis of empty BW, observed differences between forearm circumference of A × B calves and J ET and H ET calves could have been minimized. Regardless, greater forearm circumference of J ET and H ET calves compared to A × J and A × H calves was consistent with the hypothesis that beef-type genetics produce more muscular phenotypes than dairy-type genetics.

### Model to Predict Weight

The relationship between body measurements and BW has been explored in both calves and feeder cattle. Measurements of heart girth have consistently had the strongest relationship to BW and measurements of hip width consistently have the second strongest relationship (Tatum et al., 1986b; Hewitt et al., 2020). Since hip width was minimally related to frame size (Tatum et al., 1986b), using hip width to predict BW could offer value across breed types. Additionally, hip width is more conveniently measured than heart girth in most modern cattle handling systems. In the current model, most of the variation in BW was explained by the marginal effect of hip width. This exceeded the fit of previous models (driven by all calves being half siblings) and likely overestimated the ability of hip width to predict body weight in the population of terminally bound calves born on dairies. Regardless, hip width could be used in the absence of a scale to approximate calf growth in the dairy production system.

### Implications

The dairy production system limited performance of beef genetics compared to the beef production system. When embryo transfer was used to make pregnancies in Holstein and Jersey cows, resulting progeny of straightbred beef genetics were more moderate in frame size and wider than half siblings with either Holstein or Jersey maternal genetics. Additionally, a greater proportion of beef genetics was identified by greater forearm circumference—an indicator of muscularity. Angus-sired calves from Holstein dams had greater growth performance than Angus-sired calves from Jersey dams. Future studies should investigate plane of nutrition for calves of beef genetics in the dairy management system and explore opportunities to add heterosis to all-beef embryos used to make terminally bound calves on commercial dairies.

## Abbreviations

ANOVA: analysis of variance
BW: body weight
MANOVA: multivariate analysis of variance
